# Identification of Novel Toxin Genes from the Stinging Nettle Caterpillar *Parasa lepida* (Cramer, 1799): Insights into the Evolution of Lepidoptera Toxins

**DOI:** 10.3390/insects12050396

**Published:** 2021-04-29

**Authors:** Natrada Mitpuangchon, Kwan Nualcharoen, Singtoe Boonrotpong, Patamarerk Engsontia

**Affiliations:** 1 Princess Maha Chakri Sirindhorn Natural History Museum, Prince of Songkla University, Hat Yai, Songkhla 90110, Thailand; natrada.p@psu.ac.th; 2 Division of Biological Science, Faculty of Science, Prince of Songkla University, Hat Yai, Songkhla 90110, Thailand; kwan.n@psu.ac.th (K.N.); singtoe.b@psu.ac.th (S.B.)

**Keywords:** caterpillar envenomation, urticating dermatitis, venom evolution, venomics

## Abstract

**Simple Summary:**

Many caterpillar species can produce toxins that cause harmful reactions to humans, varying from mild irritation to death. Currently, there is very limited knowledge about caterpillar toxin diversity, because only a few species have been investigated. We used the transcriptome technique to identify candidate toxin genes from the nettle caterpillar *Parasa lepida* (Cramer, 1799). It is a common pest of oil palm, coconut, and mango in South and South-East Asia, which can cause severe pain and allergic responses to those in contact with them. We reported 168 candidate toxin genes. Most of them are members of the toxin genes families commonly recruited in animal venoms such as serine protease and serine protease inhibitors. However, we identified 21 novel genes encoding knottin-like peptides expressed at a high level in the transcriptome. Their predicted 3D structures are similar to neurotoxins in scorpion and tarantula. Our study suggests that *P. lepida* venom contains diverse toxin proteins that potentially cause allergic reactions and pain. This study sheds light on the hidden diversity of toxin proteins in caterpillar lineage, which could be future fruitful new drug sources.

**Abstract:**

Many animal species can produce venom for defense, predation, and competition. The venom usually contains diverse peptide and protein toxins, including neurotoxins, proteolytic enzymes, protease inhibitors, and allergens. Some drugs for cancer, neurological disorders, and analgesics were developed based on animal toxin structures and functions. Several caterpillar species possess venoms that cause varying effects on humans both locally and systemically. However, toxins from only a few species have been investigated, limiting the full understanding of the Lepidoptera toxin diversity and evolution. We used the RNA-seq technique to identify toxin genes from the stinging nettle caterpillar, *Parasa lepida* (Cramer, 1799). We constructed a transcriptome from caterpillar urticating hairs and reported 34,968 unique transcripts. Using our toxin gene annotation pipeline, we identified 168 candidate toxin genes, including protease inhibitors, proteolytic enzymes, and allergens. The 21 *P. lepida* novel Knottin-like peptides, which do not show sequence similarity to any known peptide, have predicted 3D structures similar to tarantula, scorpion, and cone snail neurotoxins. We highlighted the importance of convergent evolution in the Lepidoptera toxin evolution and the possible mechanisms. This study opens a new path to understanding the hidden diversity of Lepidoptera toxins, which could be a fruitful source for developing new drugs.

## 1. Introduction

Various animal species have evolved venoms used primarily for defense, predation, and competition [1,2]. Venoms are usually a cocktail of diverse peptides, proteins, and other organic compounds, which have multiple physiological effects on the recipients’ body as a result of millions of years of natural selection [3]. Venom toxins have long been a subject of interest due to their effects on human health, such as causing severe pain, allergic reactions, organ system failure, and death [4,5]. In contrast, many of them have potential pharmaceutical (e.g., anti-cancer, antimicrobial, neurodegenerative disorder treatment, and analgesic drugs) and pest control applications (e.g., insecticide for agriculture and mosquito-killing) [6,7,8,9,10,11].

Lepidoptera (moth and butterfly), with more than 150,000 described species, represent another lineage of animal that can produce toxins [12], of which more than 70 species from 15 families are medically important, which cause varying adverse effects to humans [13]. Unlike other venomous insects, the caterpillar does not have a specialized venom gland [14]. The secretory epithelial cells synthesize venoms and accumulate them inside the hollow canal of the injecting organs, such as urticating hairs, spines, and bristles [15,16,17]. These organs usually have sharp tips that can pierce the predators’ skin, breaking and releasing venom into the contact area [2,18]. Caterpillar venom varies in its effect from mild to severe. Local effects include contact dermatitis, e.g., allergic reaction, edema, erythema, and local tissue damage; systemic effects include hemostatic disturbance, acute kidney injury, intracerebral bleeding, and pararama-associated phalangeal periarthritis [15,18,19,20].

Currently, knowledge about the diversity and evolution of Lepidoptera toxins is still under-represented. The giant silkworm moth, *Lonomia obliqua* (Saturniidae), has been comprehensively studied most due to its venom’s lethal effects. The essential toxin proteins are Lopap (prothrombin activator) and Losac (factor X activator) proteins, which interfere with blood coagulation, leading to hemorrhagic syndrome [21,22,23]. Proteomic approaches and enzymatic assays have been applied to study toxins from other caterpillars from family taxa, including Saturniidae (*Lonomia* *achelous*, *Leucanella memusae*) [22,23], Limacodidae (*Lotoia consocia*) [16], Lymantriidae (*Euproctis chryssorrhoea*, *E. subflara*) [24], Notodontidae (*Thaumetopoea pityocampa*) [25], Erebidae (*Premolis semirufa*) [26,27], and Megalopygidae (*Podalia* ca. *fuscescens*) [23]. Various toxin classes, including serine proteases, C-type lectin, phospholipase A2, hyaluronidase, and protease inhibitors, were reported. In *T. pityocampa*, the major toxin proteins are IgE-mediated allergens, including unrelated proteins Tha p 1 and Tha p 2 [25,28]. Further information on the Lepidoptera toxin can be found in the previous excellent review [18].

We noted that the enzymatic assay used in most previous studies could not reveal the true diversity of toxins in each caterpillar species, because this method selects only a few candidate enzymes for the analysis. Recently, high-throughput sequencing technology, i.e., RNA sequencing, has been applied to study toxin genes in *Premolis semirufa* and identify more than 400 putative toxin genes [27]. This technique also provides the DNA and protein sequence and expression level of the candidate toxins genes. Thus, RNA sequencing is a promising tool to study Lepidoptera toxin diversity.

**Figure 1:**
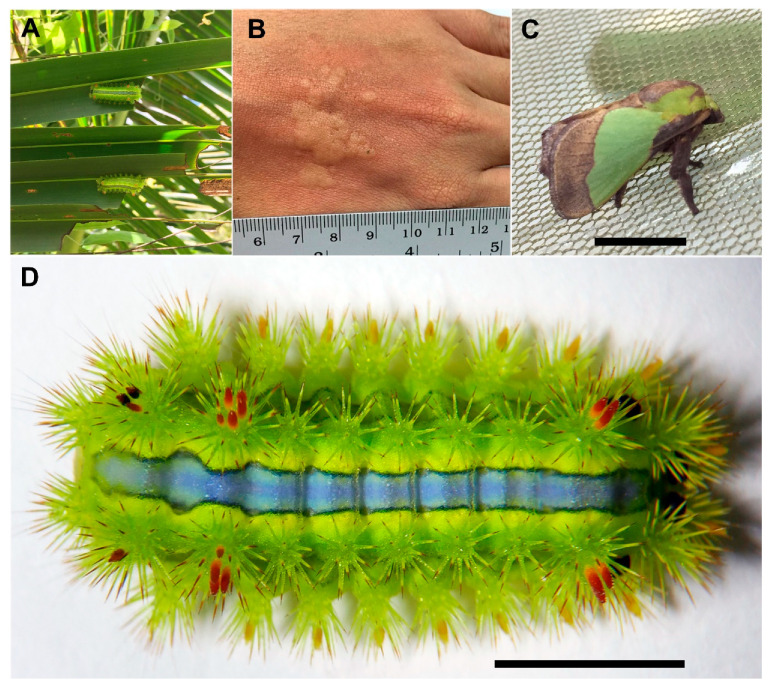
The stinging nettle caterpillar, *Parasa lepida*. (**A**) Caterpillars feeding on coconut leaves, (**B**) dermatitis symptom on a hand after exposure to caterpillar, (**C**) adult stage, and (**D**) final instar caterpillar with urticating hairs on its back (scale bar = 1 cm).

The stinging nettle caterpillar, *Parasa lepida*, is a common pest in South-East Asia, South Asia, and Southern China that feeds on leaves of oil palm, coconut, and mango trees [29,30]. The caterpillar possesses urticating hairs protruding from the dorsal side along its body’s longitudinal axis (Figure 1). It is recognized as one of the most harmful caterpillars in Thailand, causing intense pain, burning and itching sensations, and severe dermatitis [18]. Despite its adverse effects on the health of many farmers, its toxin has not been investigated. Similarly, little is known about toxins from other genera of Limacodidae, e.g., *Acharia*, *Adoneta*, *Darna*, *Euclea*, *Isa*, *Thosea*, *Phobetron*, and *Sibine* [18].

This study aimed to identify putative toxin genes from the urticating hair transcriptome of the *P. lepida* using the RNA-sequencing technique and to investigate some aspects of the gene family evolution. Our findings serve as primers to understand the hidden diversity and the evolution of lepidopteran toxin genes.

## 2. Materials and Methods

### 2.1. Caterpillars and RNA Isolation

The *Parasa* *lepida* caterpillars were collected from a local coconut farm in the Tepa district, Songkhla province, Thailand. They were brought to the laboratory and fed with fresh coconut leaves until used. To isolate the total RNA from the venom glands, final instar caterpillars were anesthetized in dry ice and placed under a stereomicroscope. The urticating hairs on the dorsal side (all clusters) (Figure 1D) were dissected at the bases. The total RNA was isolated from these tissues (~100 mg) using GENEzol^TM^ reagent (Geneaid) following the manufacturer’s protocol. RNA quantity and its integrity were assessed using NanoDrop^TM^ and gel electrophoresis, respectively.

### 2.2. RNA Sequencing and Transcriptome Analysis

The RNA sample was sent to BGI TECH SOLUTIONS (Hongkong) for the cDNA library preparation and high-throughput sequencing. In brief, RNA quality was re-evaluated using an Agilent 2100 Bioanalyzer (concentration ~ 800 ng/µL; RIN = 7.3). The mRNA was selected using magnetic beads with Oligo (dT) and fragmented using a fragmentation buffer. The cDNA was synthesized using the RNA fragments as templates and was then ligated with adapters. Sequencing was carried out on an Illumina HiSeq^TM^ 4000 (paired-ended reads, 2 × 100 bp).

Low-quality reads (more than 20% of the base’s qualities are lower than 10, N bases more than 5%, reads with adapters) were removed using Trimmomatic v.0.36 [31]. Cleaned reads were used for de novo assembly using Trinity v.2.0.6 [32]. To remove redundant sequences, assembled transcripts were clustered to a final set of Unigenes using TGICL v.2.0.6 [33]. The Unigenes are divided into two types; one type is a cluster with the prefix “CL”, followed by the cluster-id (each cluster may have several Unigenes with a similarity between them being more than 70%). Another type, the singleton, has the prefix “Unigene”.

Gene annotation of the whole transcriptome was conducted using Blast2GO v.2.5.0 [34] based on BLAST search against nucleotide and protein databases (NT, NR, KOG, KEGG, and SwissProt), gene ontology annotation, and InterPro annotation. To assess expression levels of each Unigene, cleaned reads were mapped to Unigene using Bowtie2 v.2.2.5 [35]. Expression levels were calculated into FPKM units using RSEM v.1.2.12 [36]. The full parameters used to run each program are summarized in Supplementary Data S1.

### 2.3. Identification of Toxin Genes

**Figure 2:**
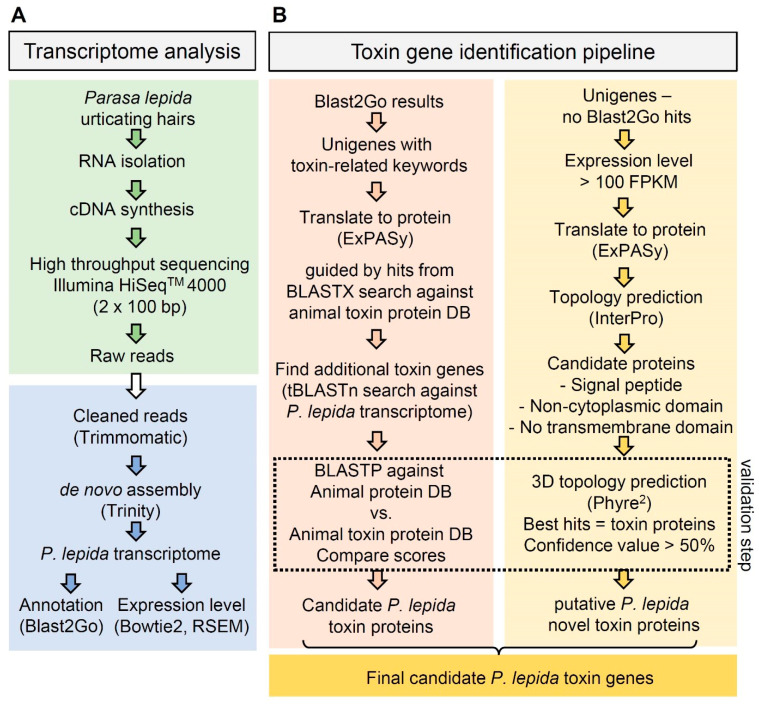
Flow chart of the *P. lepida* toxin gene identification pipeline: (**A**) transcriptome analysis—green box = RNA isolation and sequencing, blue box = transcriptome analysis; (**B**) toxin gene identification pipeline: identification based on orthologous relationships (**left**) and identification of novel toxin genes (**right**).

From the Blast2GO results, Unigenes with toxin-related keywords in the BLAST hits or InterProt prediction, e.g., lopap, serine protease, protease inhibitor, serpin, venom, toxin, allergen, CAP, and phospholipase A2 [37], were selected as candidate *P. lepida* toxin genes. These Unigenes were used as queries for the BLASTX search against our custom toxin protein database (13,124 proteins, in total), containing animal toxin proteins from the UniProt database (Animal toxin annotation project; both reviewed and nonreviewed—downloaded on the 1 May 2018), recently reported toxin proteins from Crustacea [38], and toxin proteins from *Lonomia obliqua* [21,39]. BLAST results were used to identify the translation frame of *P. lepida* Unigenes. DNA sequences were translated to proteins using the ExPASY translation tool (https://www.expasy.org (accessed on 5 May 2018)). Proteins from the correct translation frame were selected. The completeness of proteins was noted using a suffix in the gene name: Full = full gene model, NTE = N terminus missing, CTE = C terminus missing, and NC = N and C terminus missing. These candidate toxin proteins were used as queries for the tBLASTn search against the *P. lepida* transcriptome to find additional candidate toxin genes that have not been identified by Blast2GO. Additional proteins were added to the candidate toxin dataset of *P. lepida*. To remove potentially nontoxic orthologs, all candidate *P. lepida* toxin proteins were used as queries for the local-BLASTP search (e-value = 1 × 10^−5^) against two databases: (1) animal-reviewed proteins in the UniprotDB (558,125 proteins—downloaded on the 8 October 2018) and (2) our custom toxin protein database, as explained previously (Figure 2). Unigenes were filtered out from the candidate toxin gene dataset if the BLAST score of the best hit from the animal protein database was higher than the animal toxin database results. However, if the best hits were from venomous or poisonous animals, the sequences were still kept in the dataset. This guarantees that the best BLAST hits for all candidate *P. lepida* toxin proteins were known toxin proteins or proteins from venomous and poisonous animals.

All candidate toxin proteins of *P. lepida* were then separately evaluated by the protein family. They were aligned with proteins in the same family from other arthropod species (Supplementary Data S2) using MAFFT v.7. Proteins from *P. lepida*, without a conserved region, were discarded. Some proteins from two Unigenes were merged if they showed overlapping regions in the alignment, and the suffix ‘Join’ was added to the gene name. The important motifs and signatures of each protein were confirmed by InterProScan [40]. Candidate toxin genes were finally confirmed by phylogenetic relationships with previously reported toxin proteins.

To further identify putative novel toxins in *P. lepida*, which do not show significant similarity to any known protein, we selected 217 Unigenes from the *P. lepida* transcriptome that do not have hits to any database (nonredundant, nt, SwissProt, KEGG, InterPro, and GO) but have relatively high expression levels (within the top 2.5% of the highest expressed genes; expression level ranging from 100 to 24,515 FPKM) for the screening. Each sequence was inspected manually. The DNA sequences were translated using the ExPASy Translate tool, and protein domains were predicted using InterproScan. Proteins with venom protein signatures, including signal peptides, the noncytoplasmic domain, and without a transmembrane domain, were kept. These proteins were submitted to Phire^2^ (Protein Homology/analogy Recognition Engine V. 2.0) for the 3D structure prediction. Proteins were considered as putative novel toxins of *P. lepida* if their predicted 3D structures were most similar to the reported toxin proteins with a confidence value higher than 50%.

### 2.4. Gene Orthology and Phylogenetic Analysis

We analyzed whether some families of *P. lepida* candidate toxin genes have undergone expansion or contraction by comparing the number of orthologous genes from other Lepidoptera species. To identify orthologous genes, we used OrthoVenn2 [41] to search against available lepidopteran genomes (*Danaus plexippus*, *Heliconious melpomene*, *Malitaea cinxia*, and *Bombyx mori*) and a toxin gene dataset from *Lonomia obliqua* [21,39] using *P*. *lepida* genes as queries (E-value = 1 × 10^−5^; inflation value = 1.5). Then, we used the chi-square test to examine whether the number of genes in some toxin gene families of *P. lepida* deviates from a 1:1 ratio, which indicates gene family expansion or contraction.

Phylogenetic analyses of individual toxin families were conducted to reconstruct the evolutionary relationship of these toxin proteins within the arthropod lineage. *P. lepida* proteins were aligned with toxin proteins from other arthropods, including Chelicerata, Myriapoda, Crustacea, and other insects retrieved from the NCBI UniProt database (Supplementary Data S2). Sequence alignment was carried out using MAFFT version 7.0 followed by removing gappy regions using TrimAl (http://trimal.cgenomics.org (accessed on 5 May 2018)). Trees were made using PhyML with Akaike Information Criterion automatic model selection [42]. Support values for branches were analyzed using 1000 replications bootstrapping. Trees were illustrated by FigTree version 1.4.4 (http://tree.bio.edu.ac.uk.software.figtree (accessed on 5 May 2018)).

## 3. Results

### 3.1. Transcriptome Assembly and Gene Annotation

Illumina HiSeq4000 sequencing generated about 68 million reads and 6.8 billion bases in total (Table 1). After de novo assembly and filtering the redundant sequences, we received 34,968 Unigenes for the downstream analyses. The total length, average length, N50, and GC content of the analyzed Unigenes were 27,807,171 bp, 795 bp, 1315 bp, and 36.73%.

**Figure 3:**
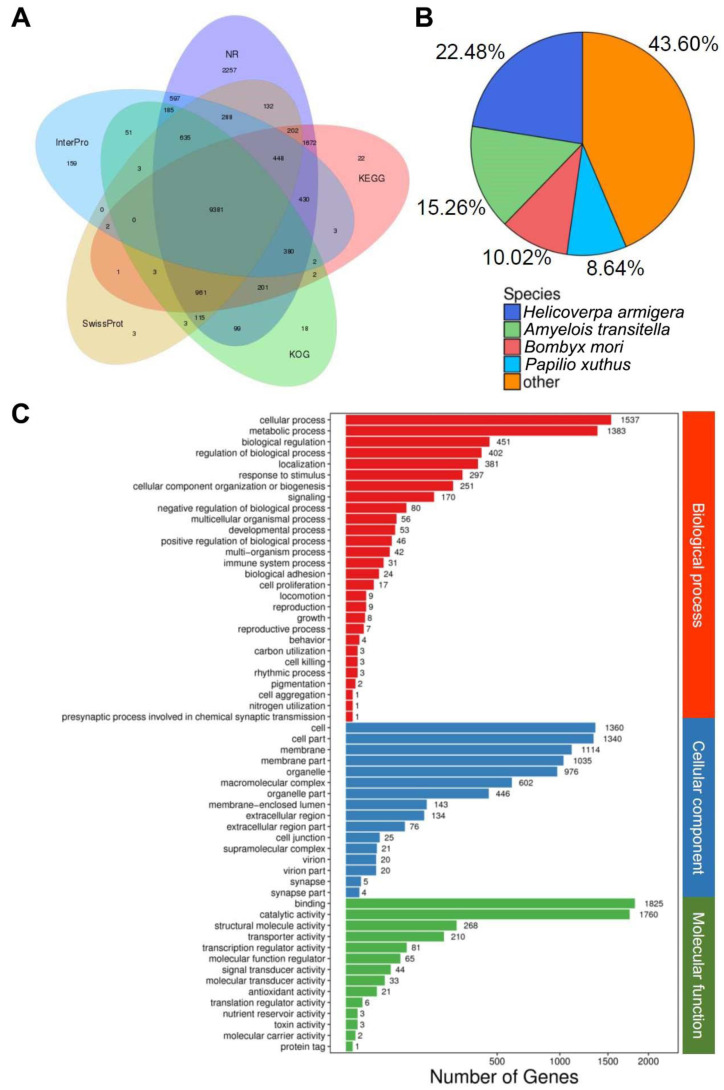
Overview of the BLAST2GO results: (**A**) intersection of different databases and numbers of contigs, (**B**) species distribution of best BLAST hits, and (**C**) genes classified by different categories (biological process, cellular component, and molecular function) and number of genes.

All Unigenes were annotated by searching against seven available databases using Blast2GO (BioBam^®^). In summary, 17,983 (51.43%) and 6175 Unigenes (17.66%) were annotated from nonredundant nucleotide and protein databases from NCBI, respectively. In addition, 12,177 (34.82%), 12,039 (34.43%), 13,710 (39.21%), 4107 (11.75%), and 12,564 (35.93%) were annotated from Swissprot, KOG, KEGG, and InterPro databases, respectively (Figure 3A). Species of the best matching BLAST hits were *Helicoverpa armigera* (22.48%), *Amyelois transitella* (15.26%), and *Bombyx mori* (10.02%) (Figure 3B).

Gene ontology analysis indicated the expression of diverse proteins in the urticating hairs of the *P. lepida*. They were classified into three main categories: biological process, cellular component, and molecular function. The top three subgroups for each category in terms of the number of genes were as follows: (1) cellular process, metabolic process, biological regulation (biological process) (1537, 1383, and 451 Unigenes, respectively); (2) cell, cell part, and membrane (cellular component) (1360, 1340, and 1114 Unigenes, respectively); and (3) binding, catalytic activity, and structural molecular activity (molecular function) (1825, 1760, and 268 Unigenes, respectively) (Figure 3C). The full annotation results of the *P. lepida* urticating hair transcriptomes are reported in Supplementary Data S3.

### 3.2. Candidate Toxin Genes of Parasa lepida

Following our toxin gene identification pipeline, we reported 168 candidate toxin genes classified into four main groups and 15 subgroups. The first three main groups were conventional toxin classes that share homologies with known toxins from other venomous animals: (1) protease inhibitor (lipocalin, serpin, Kazal-type protease inhibitor, Kunitz-type protease inhibitor, and trypsin inhibitor), (2) proteolytic enzyme (peptidase S1, peptidase S10, and venom dipeptidyl peptidase-4), and (3) other toxins (CAP (CRISPs, Ag5, and Pr-1), C-type lectin, phospholipase A2, venom carboxyesterase6, venom acid phosphatase, and antimicrobial peptides). The fourth group involved the candidate novel toxin proteins from *P. lepida* that do not share homologies to any sequence in the NCBI database. All of these proteins contain a Knottin-like domain, thus referred to here as a Knottin-like peptide. Full data of these candidate toxin proteins are reported in the Supplementary Data S4.

**Figure 4:**
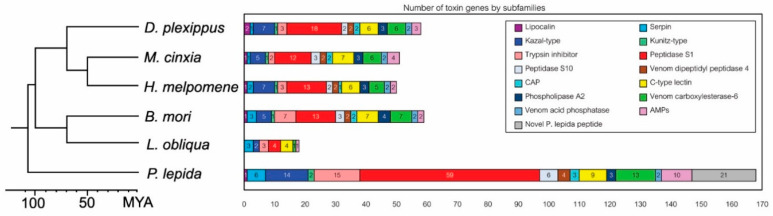
Number of toxin genes by subfamilies of *P. lepida* and the number of their orthologous genes presented in other Lepidopteran genomes (*Danaus plexippus*, *Melitaea cinxia*, *Heliconius melpomene*, and *Bombyx mori*) and cDNA library (*Lonomia obliqua*).

The largest toxin group was a serine protease, which has 59 genes in total, accounting for 35% of all toxin genes identified in this study, followed by the *P. lepida* Knottin-like peptide (21 genes), trypsin inhibitor (15 genes), Kazal-type protease inhibitor (14 genes), venom carboxylesterase-6 (13 genes), antimicrobial peptide (10 genes), C-type lectin (9 genes), peptidase S10 (6 genes), serpin (6 genes), venom dipeptidyl peptidase 4 (4 genes), CAP (3 genes), phospholipase A2 (3 genes), venom acid phosphatase (2 genes), Kunitz-type protease inhibitor (2 genes), and lipocalin (1 gene) (Figure 4).

### 3.3. Gene Family Expansion

We identified orthologous genes of the *P. lepida* candidate toxin genes in other Lepidopteran species using OrthoVenn2 (E-value = 1 × 10^−5^; inflation value = 1.5) (Figure 4 and Supplementary Data S5). The number of genes for each toxin gene family varied between species; however, comparing the four species with genome data, the number of orthologous genes was in a ratio of about 1:1 for all gene families. The number of genes in *L. obliqua* was fewer than in other species, most possibly due to incomplete annotation. The number of genes in subfamily Peptidase S1 in *P. lepida* was much higher (59 genes) than in other species (12–18 genes), suggesting lineage-specific expansion. We performed a chi-square test on gene subfamilies with more than five genes in all species (Venom carboxylesterase-6, C-type lectin, and Peptidase S1). The number of genes in the peptidase S1 subfamily significantly deviated from the 1:1 ratio (X^2^ = 71.391, df = 4, *p*-value < 0.0001), but this was not the case for venom carboxylesterase-6 and C-type lectin (X^2^ = 5.568, df = 4, *p*-value = 0.2339; X^2^ = 0.857, df = 4, *p*-value = 0.9306, respectively).

**Figure 5:**
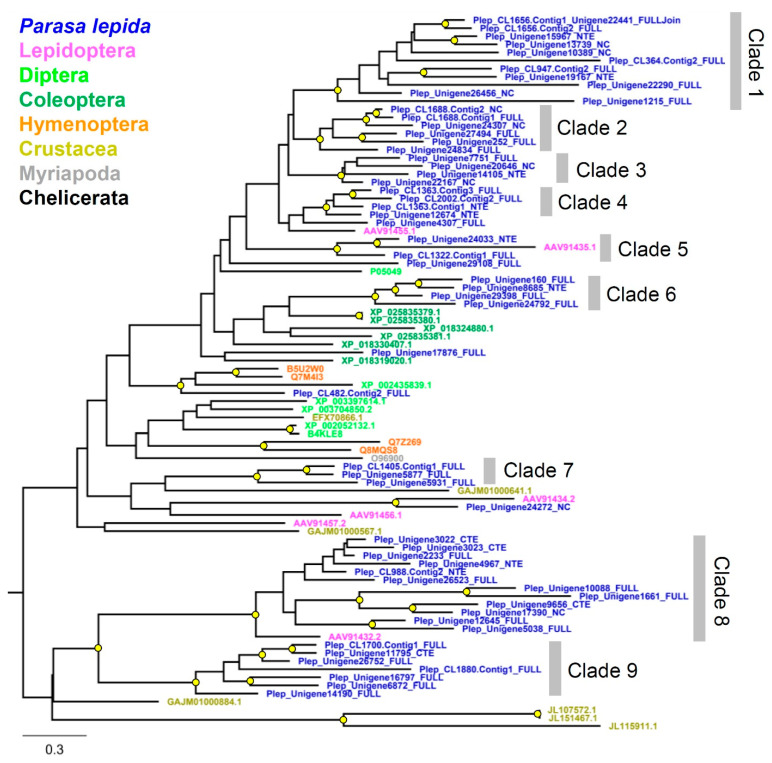
Phylogenetic tree of the serine protease gene family from *Parasa lepida* and other Arthropods. Clades were defined based on phylogenetic relationships with branch support. The tree was constructed using the maximum likelihood method by PhyML 3.0. A branch support value higher than 70% (1000 bootstrap replications) is shown with a yellow circle. The tree was rooted at the midpoint.

Phylogenetic relationships of *P. lepida* Peptidase S1 genes revealed multiple lineage-specific expansions (Figure 5), explaining this subfamily’s large size. Genes are divided into nine clades. The expansions were observed in clades 1, 8, and 9 (11, 12, and 7 genes, respectively). These genes’ closest relatives are serine proteases from *L. oblique* (AAV91435.1, AAV91432.2, and AAV91434.2).

### 3.4. The Expression Level of the Candidate Parasa lepida Toxin Genes

**Figure 6:**
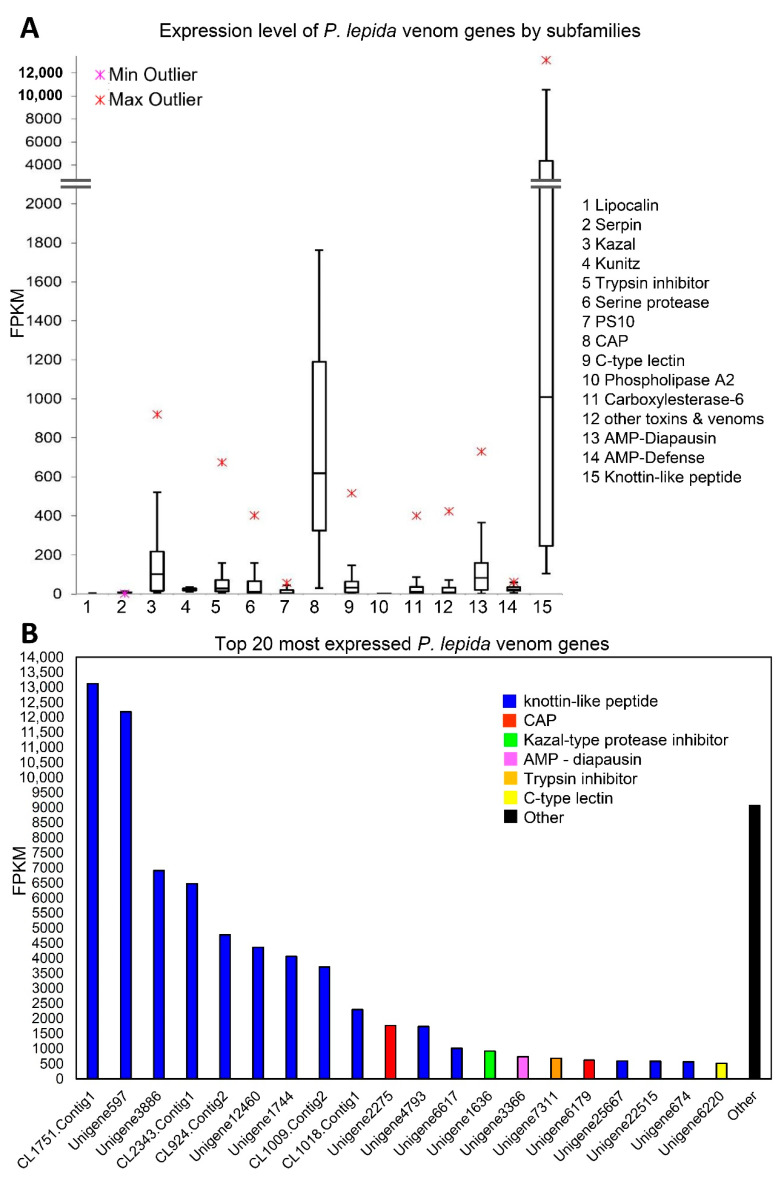
(**A**) Box-and-Whisker plot showing expression level (FPKM) of *P***.** *lepida* venom genes by subfamily. Boxes show the upper quartile and lower quartile. The line across the box indicates the median. The maximum and minimum values (excluding outliers) are shown above and under the box, respectively. Upper and lower outliers are indicated using red and pink symbols, respectively. (**B**) The expression level of the top 20 most expressed *P. lepida* toxin genes.

Expression levels of genes were estimated by mapping reads to assembled contigs. The numbers of mapped reads (counts) was normalized by contig length and total mapped reads. The expression levels among candidate toxin genes varied between 0.38 and 13,119.23 FPKM (Fragments Per Kilobase of transcript per Million mapped reads). The top three most expressed families (according to median) were Knottin-like peptides, CAP, and Kazal (Figure 6A). Interestingly, nine out of ten most expressed toxin genes were novel Knottin-like peptides (Figure 6B). The expression levels of the 21 novel Knottin-like toxins were also within the top 0.5% most expressed genes in the *P. lepida* urticating hair transcriptome. Other genes in the top 20 were CAP, Kazal-type protease inhibitor, AMP-diapausin, trypsin inhibitor, and C-type lectin (Figure 6B).

### 3.5. Putative Novel Toxin Proteins from P. lepida

**Figure 7:**
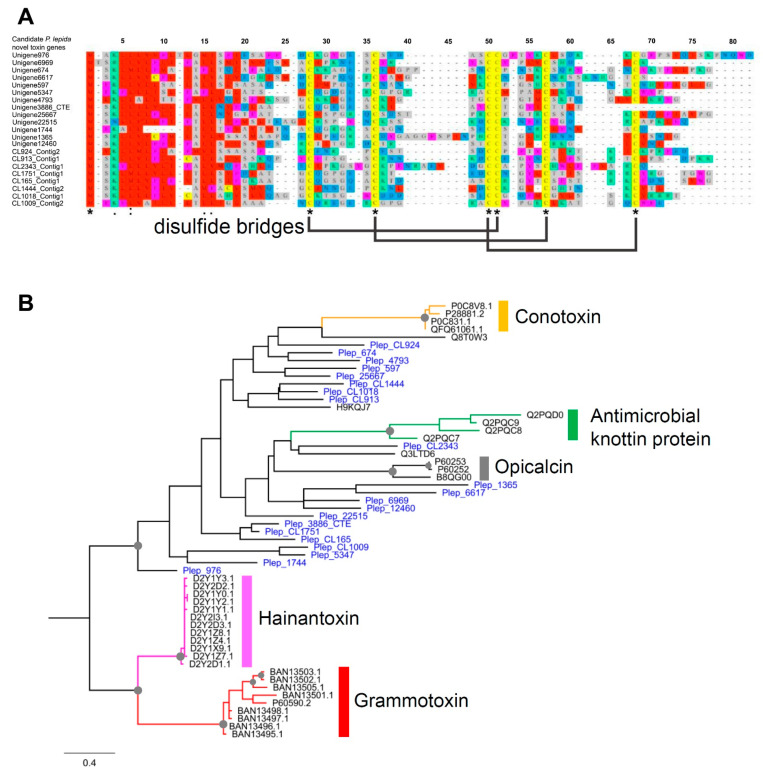
(**A**) Protein alignment of novel Knottin-domain protein from *P. lepida* showing six conserved cysteine residues and putative three disulfide bridges; (**B**) phylogenetic tree of Knottin-like proteins from *P. lepida* and other animals: Conotoxin—*Conus achatinus* (Little frog cone), Antimicrobial Knottin protein—*Bemisia tabaci* (Silverleaf whitefly), Opicalcin—*Opistophthalmus carinatus* (African yellow leg scorpion), Hainantoxin—*Cyriopagopus hainanus* (Chinese bird spider), Grammotoxin—*Grammostola rosea* (Chilean rose tarantula). Branch supports higher than 70% (1000 replications bootstrapping) are highlighted with grey circles.

We identified 21 novel toxin peptides from *P. lepida*. Either DNA or protein sequence shares a similarity to known sequences in the NCBI database. Their sizes vary from 46 to 71 amino acids ($\bar{x}$ ± SE = 56 ± 1.19). These proteins’ pairwise distances revealed a wide range of sequence diversity as the percent identity varies between 17.33 and 73.33%. These proteins have a signal peptide and a noncytoplasmic domain. Protein alignment revealed six conserved cysteine residues (Figure 7A). These conserved domains were predicted from the three disulfide bridges structure of the inhibitor cystine knot motif. Phire2 (Protein fold recognition server) predicted the 3D structures of these proteins similar to the neurotoxins from spiders, scorpions, and cone snails such as hptx2, ptu-1, hainantoxin-iii, actx-2, and omega-conotoxin (Supplementary Data S4).

Phylogenetic analysis of these 21 *P. lepida* novel toxins and other Knottin domain-containing toxins from other animals, including spiders, scorpions, insects, and cone snails, revealed nonmonophyletic relationships of *P. lepida* proteins (Figure 7B). These proteins are more likely to arise from convergent evolution based on sequence divergence and unclear relationships with other animals’ toxins. Besides, the long branches in a phylogenetic tree suggested that they are not the product of recent gene duplication or lineage-specific expansion.

## 4. Discussion

### 4.1. Predicted Functions of P. lepida Toxins

#### 4.1.1. Knottin-Like Peptides

We predicted that Knottin-like peptides play significant roles in the *P. lepida* venom, and they are neurotoxins. This is because they showed the highest expression genes among *P. lepida* toxin gene families (Figure 6) and were unique to *P. lepida,* suggesting that they are the product of the lineage-specific adaptation in *P. lepida* venom evolution. Their predicted 3D structures are similar to Knottin-containing domain neurotoxins from distantly related animals (Supplementary Data S4). For example, conotoxins from cone snail and grammotoxins from tarantula block voltage-gated Ca^2^^+^ channels and cause paralysis [43,44]. Other neurotoxins are hainantoxins from the spider that can inhibit voltage-gated Na^2^^+^ channels, and opicalcins from the scorpion that can activate Ca^2^^+^ release channel/ryanodine receptors, which also cause paralysis [45,46,47]. Knottin proteins (δ-theraphotoxin-Hm1a and -Hm1b) in *Heteroscodra maculate* tarantula venom activate the voltage-gated Na^2^^+^ channels nociceptor, which elicits the pain sensation [48]. A recent study demonstrated that short peptides from the *Parasa* *(Latoia) consocia* caterpillar play essential roles in the caterpillar venom by activating the nociceptive ion channel TRPV1, which causes the burning pain sensation [16]. However, the proteins have not yet been fully characterized, so we cannot conclude whether they are orthologous proteins. Further investigations are required to confirm whether the novel Knottin-like proteins play a crucial role in *P. lepida* venom by activating the recipients’ pain receptors. Interestingly, a recent transcriptomic study in the Brazilian moth, *Premolis semirufa*, also identified putative knottin-like toxins (0.7% of all candidate toxins), which have protein sequences similar to neurotoxins from spiders [27]. This may reflect the convergent evolution of knottin-like toxins in venomous Lepidoptera, warranting further investigation.

#### 4.1.2. CAP Family

The second-highest expression belongs to the CAP family (Figure 6). This family has three subclasses: Cysteine-rich secretory proteins (CRISPs), Antigen 5 (Ag5), and Pathogenesis-related 1 proteins (Pr-1) [49]. All of the three genes identified from *P. lepida* are in the Ag5 subclass. In Hymenoptera, CAP proteins are the primary allergen that activates IgE, causing an extreme allergic reaction [50,51,52]. In snake venom, CRISPs inhibit smooth muscle contraction [53], and in cone snail venom, CRISPs act as protease [54].

#### 4.1.3. Venom Dipeptidyl Peptidase IV

Venom dipeptidyl peptidase IV is an enzyme that removes N-terminal dipeptide from the polypeptide [55]. We identified four venom dipeptidyl peptidase IV genes from the *P. lepida*. Their roles include activating an immune response, initiating the synthesis of antimicrobial peptides, and activating immune cells’ movement [55,56]. This protein is a significant allergen in *Vespa vulgaris* and *Polistes dominula* venom and it has been reported in other animal venoms, including snakes, bees, and ants [55,57,58].

#### 4.1.4. Venom Acid Phosphatase

Venom acid phosphatase hydrolyzes phosphomonoester at acidic pH. We identified two venom acid phosphatase genes in the *P. lepida* transcriptome. It is a significant allergen in bee venoms that can induce wheal and flare reactions in humans by releasing histamine [59,60]. They can also activate cell histolysis and tissue degeneration [61]. They are found in various insects, particularly from the order Hymenoptera [62,63,64].

#### 4.1.5. Carboxylesterase-6

Carboxylesterase-6 is a hydrolyzing enzyme that cuts carboxylic esters. We identified 13 carboxyl esterase-6 genes in the *P. lepida* transcriptome. Carboxylesterase-6 is the significant allergen in hymenopteran insects’ venoms [59,65,66,67]. They also have roles as an odorant degrading enzyme in the insect olfactory sensilla [68].

#### 4.1.6. Serine Proteases

Serine proteases may also play essential roles in *P. lepida* venom, as observed from a large number of genes (59 genes), which is about four times higher than the number of orthologous genes shared among lepidopteran species (14 genes) (Figure 4). Serine proteases are the most common class of proteins presented in venoms of various animals, including many arthropods (e.g., centipede, Remipedia, bee, wasp, and tick), mollusk, annelid, and vertebrates (e.g., reptile, platypus, and vampire bat) [37,38,69,70,71,72,73]. They have diverse physiological effects, including blood coagulation, blood vessel relaxation, contraction of smooth muscles, initiation of pain, suppression of immunity, inflammation, and melanization [74,75].

#### 4.1.7. Lipocalins

Lipocalins are small extracellular proteins that form a large protein family. We identified one candidate *P. lepida* lipocalin toxin gene. Lipocalins have diverse physiological roles, including transporter (retinol, odorant, and pheromone), color mimicry, prostaglandin synthesis, cell homeostasis, and immunity [76]. Some of them are allergenic proteins causing severe anaphylaxis in humans [77]. They also function as an anticoagulant in the toxins of snakes, bats, and the saliva of ticks, kissing bugs, and mosquitoes [3,37,78,79]. Lopap of *L. oblique* is a lipocalin with serine protease properties. It functions as a prothrombin activator, which causes a severe consumptive coagulopathy [80].

#### 4.1.8. Serine Protease Inhibitor (Serpin)

Serpins inhibit serine protease by permanently altering its structure, and they can inhibit other enzymes such as caspases and papain-like cysteine protease [81]. This study reported six serpin genes in the *P. lepida* transcriptome. Serpin is a large and diverse protein family and it is present in the venom of *Lonomia obliqua*, *Leucanella memusae*, and *Podalia* ca. *fuscescens* caterpillar [23]. Serpin regulates protein digestion in the crucial biological process, e.g., blood coagulation, inflammation, and immune response [37,81]. Serpins are present in many animal venoms, including platypus, snake, cnidarian, leech, Remipedia, centipede, caterpillar, wasp, tick, and mosquito [37,81,82,83].

#### 4.1.9. Kazal-Type Protease Inhibitor

The Kazal-type protease inhibitor is a serpin containing one or more Kazal domains. These proteins inhibit serine protease, e.g., thrombin, trypsin, factor XIIa, subtilisin A, elastase, chymotrypsin, and plasmin [84]. We identified 14 Kazal-type protease inhibitors from *P. lepida*. Kazal-type protease inhibitors are present in the venoms of snake, bat, leech, jellyfish, tick, and many insects, e.g., caterpillar, termite, bee, wasp, mosquito [37,74,83,85].

#### 4.1.10. Kunitz-Type Protease Inhibitor

The Kunitz-type protease inhibitor is a serpin containing the Kunitz domain. We identified two Kunitz-type protease inhibitor genes from *P. lepida*. These proteins can inhibit trypsin, chymotrypsin, as well as potassium and voltage-gated sodium channels [86,87], which interfere with biological activities, such as blood coagulation, inflammation, and pain suppression [86,88,89]. This protein is also present in the venom of snake, frog, leech, sea anemone, cone snail, scorpion, spider, tick, and many insects, including bees and wasps [37,90,91].

#### 4.1.11. Trypsin Inhibitor

The trypsin inhibitor is another serpin that inhibits trypsin, which functions as an irreversible competitive substrate and can impede blood coagulation [92]. We identified 15 trypsin inhibitor genes from *P. lepida*. They occur in the venom of *L. obliqua* [21] and scorpions, e.g., BmKAPi in *Tityus bahiensis*, *T. obscurus*, and *T. serrulatus* [92,93].

#### 4.1.12. Serine Carboxypeptidase

This protein is another member of the peptidase family, but there are few reports of serine carboxypeptidase in the animal venom than the serine protease. We identified six serine carboxypeptidase genes from *P. lepida*. They have diverse physiological roles, e.g., releasing histamine, destroying neurotransmitters, neurotoxicity, immunity, and venom proteins’ phosphorylation [94]. They are allergens in bee venoms that elicit an IgE-mediated allergic reaction [94]. Serine carboxypeptidases are found in bee, wasp, kissing bug, and Remipedia venoms [37,69,70,71,72,73].

#### 4.1.13. C-Type Lectin

C-type lectin is an extracellular carbohydrate-binding protein, including multifunctions such as cell adhesion, pathogen recognition, an inflammatory response, hemostasis, and hemagglutination [95,96]. We identified nine C-type lectin genes from the *P. lepida*. They are also present in many animal venoms, including *L. oblique*, snake, lionfish, cnidarian, crustacean, insects, caterpillars, and leech [21,37,96,97,98,99].

#### 4.1.14. Phospholipase A2

Phospholipase A2 hydrolyze various phospholipids. They can induce cell death, inflammation, edema, anticoagulation, and inhibit transmitters at the neuromuscular junction [3,96,100]. There are three putative venom phospholipase A2 genes in the *P. lepida* transcriptome. In *L. obliqua*, phospholipase A2 has an indirect hemolytic activity in human and rat red blood cells [22]. Phospholipase A2 is a potent toxin in the snake venom and is also found in the venom of other species, including reptile, cnidarian, mollusk, hymenopteran insect, scorpion, spider, and annelid [3,21,101,102].

#### 4.1.15. Antimicrobial Peptides

We identified two types of antimicrobial peptides, diapausin and attacin, from the *P. lepida* transcriptome (six and four genes, respectively). Diapausin is a cysteine-rich peptide that has an antifungal property by inhibiting α-1,3-glucan synthesis [103]. They also function as an N-type voltage-gated calcium channel blocker due to their similar structure to ω-conotoxin GVIA in cone snail toxins [104]. Diapausin is present in various arthropods, including collembola, beetles, and butterflies [105,106,107]. Attacin is a glycine-rich peptide that inhibits the growth of Gram-negative bacteria by inhibiting cell wall production [108,109,110]. They are reported in Lepidoptera and Diptera. This protein’s toxicity is unclear, but it is expressed in the venom of *L. obliqua* [21].

### 4.2. P. lepida Toxins in Comparison with Other Venomous Lepidoptera

In total, we reported 168 candidate toxin genes from the *P. lepida* urticating hair transcriptome, indicating that the toxicity of *P. lepida* venom is most likely the product of a complex toxin cocktail. Similarly, the diversity of toxins is also observed in *Premolis semirufa* caterpillar as 418 candidate toxin genes are identified from its transcriptome [27]. We note that different annotation methods partly explain the distinct difference between these numbers. The study in *Premolis semirufa* does not have a validation step to filter out some housekeeping genes from the sequences that match known toxins. For this reason, the number of genes in each toxin class between the two species cannot be compared directly. Some toxin classes of *P. lepida* are similar to those of *L. obliqua* and *Premolis semirufa*, including protease inhibitor (serpin, Kazal-type protease inhibitor, and trypsin inhibitor), proteolytic enzyme (peptidase S1), and other toxins, including lipocalin, C-type lectin, and AMPs [21,22,27]. Serine protease genes, which are greatly expanded (59 genes) in *P. lepida*, are also present in the venom of *Leucanella memusae*, *Euproctis chrysorrhoea*, *Euproctis subflara*, *Premolis semirufa*, and *Podalia* ca. *fuscescens* [23,26,27]. These species are from different taxonomic lineages, suggesting that some toxin classes, particularly serine protease, are repeatedly recruited in the Lepidoptera venom evolution.

Our transcriptome analysis did not detect closely related genes of Lopap and Losac, which are vital toxins in the *L. obliqua* venom. These proteins are prothrombin activator and factor x activator, respectively. They can interfere with the blood coagulation process, causing hemorrhagic syndrome and acute kidney injury [111,112]. The closely related species, *Lonomia achelous*, also has proteins that cause blood coagulation, e.g., Lonomin (urokinase-like), Lonomin III (prothrombin activator), Lonomin IV (Factor Xa-like), and Lonomin V (FXIII inactivator) [22]. The differences in toxin gene composition explain why the venom of *P. lepida* and *Lonomia* spp. causes different physiological effects.

Some toxin classes of *P. lepida* have not been reported in other venomous Lepidoptera but are shared with *Premolis semirufa*. These include venom peptidyl peptidase 4, venom acid phosphatase, and carboxylesterase-6 [18,27]. The putative functions of these proteins fit the effects of *P. lepida* venom, e.g., contact dermatitis. As discussed previously, the novel Knottin-like peptides are expressed at a high level and may function by activating pain receptors, causing stinging pain. Simultaneously, other proteins that are allergens similar to those found in hymenopteran venoms can cause allergic reactions in the contact area, including erythema, edema, intense itch, and inflammation.

However, we note that several aspects limit the comparison and interpretation. First, only a few venomous lepidopteran species have been investigated. Secondly, previous research did not use a high-throughput sequencing technique, e.g., transcriptome and genome; thus, the diversity of toxin genes might not have entirely be revealed. For these two reasons, the assumption that particular toxin classes are unique to a specific lepidopteran species or lineage might be incorrect. Third, *P. lepida* toxins’ functions are predicted based solely on orthologous relationships with previously reported toxins or the predicted 3D structure with known toxins. Further functional tests are required to confirm the venom activity of the *P. lepida* candidate toxins.

### 4.3. Evolution of P. lepida Toxin Genes

Many lepidopteran species from different families can produce venoms, suggesting that toxin genes evolved independently many times in Lepidoptera evolution. Convergent evolution is a fundamental process in animal venom evolution [3,113]. Our findings indicate that some toxin classes have been repeatedly recruited into the venom of Lepidoptera, particularly serine protease, protease inhibitor, phospholipase A2, and lectin, as reported in *P. lepida* and other species. The convergent evolution can also occur across animal lineage; for example, *P. lepida* and hymenopteran venoms share similar toxin compositions, including CAP, Kunitz, carboxylesterase-6, and venom acid phosphatase, which are allergens that trigger the recipient’s immune system [57].

Due to a significant expansion of serine protease genes (59 genes) and Knottin-like peptide genes (21 genes) in *P. lepida*, processes involving gene duplication, followed by positive selection, may be responsible for this. This process is one of the most critical mechanisms in venom gene evolution, as documented in snakes, cone snails, spiders, scorpions, and centipedes [82,114,115,116,117,118,119]. Tandem gene duplication may explain the gene family expansion reported in the genome of *Stegodyphus mimosarum* spiders, which has 51 Knottin-like toxin genes [120], and *Mesobuthus martensii* scorpion, which has more than 100 neurotoxin genes (NaTx, KTx, and ClTx) [121]. When the genome data of *P. lepida* are available, and genes’ locations are determined, it will confirm whether the serine protease genes of *P. lepida* arose from tandem gene duplication.

Interestingly, putative toxin genes encoding 21 Knottin-like peptides in *P. lepida* venom have no significant similarity to any previously reported gene in the NCBI database, suggesting that they may arise specifically in the ancestral lineage of *P. lepida*. Due to the limitation of its related species’ genetic data, it is impossible to fully understand the origin of these genes. Phylogenetic analysis indicates that these 21 genes are not monophyletic (Figure 7B). They also have long branches, suggesting an ancient origin, and proteins from different branches may serve different functions. We hypothesized that they might arise from the nontoxic Knottin proteins in the ancestor of Lepidoptera. Mutations of housekeeping genes may lead to toxic functions, possibly neurotoxins, favored by natural selection. This fits the theory that toxins for defense purposes should be fast-acting by interfering with nerve transmission [1]. Other reported roles of Knottin peptides are antimicrobial peptides. For example, tachystatins from horseshoe crab hemocytes provides activity against Gram-negative and Gram-positive bacteria, and fungi [122]. In whitefly *Bemisia tabaci*, Knottin-like proteins play a role in regulating the yellow leaf curl virus, and they are upregulated after infection with *Pseudomonas aeruginosa* [123,124,125].

Our results support previous observations [3] that animal toxins may arise convergently from ancestral proteins, which have the following properties: secretory protein, functionally versatile, stable molecular scaffolds, and extensive disulfide cross-links. Once recruited in animal venom, these proteins evolved under positive selection and were subject to neofunctionalization.

## 5. Conclusions

We identified 168 candidate toxin genes from the *P. lepida* transcriptome. Of these, 147 genes are from 13 toxin classes commonly found in venomous animals and 21 genes encoding novel peptides unique to *P. lepida*. We predicted that *P. lepida* toxins have diverse physiological roles, including neurotoxins that initiate intense pain (Knottin-like peptides), allergens activating the recipients’ immune system (e.g., CAP, Kunitz, carboxylesterase-6, and venom acid phosphatase), and toxins causing inflammation (e.g., serine protease, phospholipase A2, and serpin). This prediction, however, requires future functional tests. We highlighted the importance of convergent evolution in the Lepidoptera venom and possible mechanisms, such as mutation of the house-keeping gene and gene duplication, followed by neofunctionalization. We believe this study opens further investigations into toxins in Lepidoptera, the understudied group that has potential benefits in pharmacy, medicine, biotechnology, and agriculture.

## Figures and Tables

**Table 1:** Information of sequence reads generated by RNA-seq, and Trinity de novo-assembled contigs.

Sequence Analysis	Counts	Length (bp)
Raw sequence reads acquired	67,876,696	6,787,669,600
Sequence reads after QC	66,361,788	6,636,178,800
Contigs after trinity assembly	48,761	34,507,843
Unique contigs (Unigene)	34,968	27,807,171
GC content of Unigene	36.73%	
Average length		795
N50		1315

## Data Availability

RNA sequencing reads have been deposited in the Sequenced Read Archive (SRA) under BioProject ID: PRJNA694339.

## Supplementary Materials

The following are available online at https://www.mdpi.com/article/10.3390/insects12050396/s1, Supplementary Data S1. Parameter settings used in transcriptomic analyses; Supplementary Data S2. Accession IDs of the arthropod toxin proteins; Supplementary Data S3. Gene annotation results from BLAST2GO; Supplementary Data S4. *Parasa lepida* candidate toxin genes; Supplementary Data S5 Lepidoptera orthologous genes identified by OrthoVenn2.
