# Supplementary material for: Identification of Novel Toxin Genes from the Stinging Nettle Caterpillar Parasa lepida (Cramer, 1799): Insights into the Evolution of Lepidoptera Toxins
Source: Insects. 2021 Apr 29;12(5):396. doi: 10.3390/insects12050396 (PMC8145965; doi:10.3390/insects12050396)
Supplement: Supplementary file 1 [file insects-12-00396-s001.zip › insects-1193693-supplementary/Supplementary data/Supplementary data S1_RNA-seq parameters.pdf]

Supplementary data A5

Parameters setting used in transcriptomic analyses

No.	Programs	Parameters
1	Trinity v2.0.6	--min_contig_length 150 --CPU 8 --min_kmer_cov 3 --min_glue 3 --bfly_opts '-V 5 --edge-thr=0.1 --stderr'
2	Tgicl v2.0.6	-l 40 -c 10 -v 25 -O '-repeat_stringency 0.95 -minmatch 35 -minscore 35'
3	Blast2GO v2.5.0	default
4	TransDecoder v3.0.1	default
5	Bowtie2 v2.2.5	-q --phred33 --sensitive --dpad 0 --gbar 99999999 --mp 1,1 --np 1 --score-min L,0,-0.1 -I 1 -X 1000 --no-mixed --no-discordant -p 1 -k 200
6	RSEM v1.2.12	default
